# Supplementary material for: Fleeing the war: A socio-ecological perspective on the mental health of internally displaced and refugee children and adolescents living in the Kurdistan region of Iraq
Source: PLOS Ment Health. 2025 Jan 30;2(1):e0000170. doi: 10.1371/journal.pmen.0000170 (PMC12798447; doi:10.1371/journal.pmen.0000170)
Supplement: Attachment — Submitted filename: Response to Reviewers.docx [file pmen.0000170.s001.docx]

Dear Dr. Pierre Olivier Jacquet,

thank you very much for your helpful feedback, we highly appreciate the effort you have taken in reviewing the manuscript yourself. We have revised the manuscript accordingly and addressed the raised concerns as described below.

**Editor Q1)**

**For example, the selection of predictors included in regression models is not always well justified. The decision criteria used to exclude important variables (as those listed in table 1, e.g., level of education) from the regression models are missing. This is a crucial point because some of these variables could potentially increase or decrease the strength of the associations between some of the predictors included in the regression models and the outcomes.**

Thank you for raising this relevant point. Our selection of predictors included in the regression models was based on the existing literature as well as our assessment of the interpretability of the variables according to our impressions during data collection. Here are the reasons for excluding the following variables from the regression models:

- Level of education: During data collection, we noticed that the interpretability of this variable (completed years of school) is limited. As there is no functioning schooling system in the camps, the variable is much more related to factors such as age and gender, rather than reflecting actual education level within our sample. Therefore, we decided not to include it as predictor in the regression models.
- Religion and ethnicity: Based on the existing literature and to our knowledge, it may be difficult to justify the assumption that mere religious or ethnic affiliation is associated with higher mental health symptomatology. Due to a lack of scientific evidence and in order not to promote stigmatization of religious or ethnical groups, we refrained from expecting associations between mere group belongings and higher mental health symptomatology.
- Place of living during war, place of living before war: We did not include these variables as they involve retrospective information going back many years for the majority of minors since children have been living in the camp for an average of 4 years, rather than reflecting their current living situation.

**Editor Q2)**

**Furthermore, multiple testing is a potential source of false positives that is not taken into account in the current version of the manuscript. The problem of multiple testing could be offset by applying a bootstrapping procedure to the estimation of the parameters of each model (bootstrapped CIs and exact p-values would then be reported in the results).**

Thank you for your comment. We carefully considered multiple testing correction. First of all, we would like to point out that the multiple regression analyses were conducted in three different samples, accordingly, we only conducted four multiple regression analyses per sample which might require correction. Further, the outcome variables in our regression models are all facets of mental health rather than independent factors. Thus, due to the relatively high correlations between the dependent variables of the regression models belonging to one construct (mental health, see tables below), we did expect rather similar patterns in the regression models, instead of independent results. Last, the overall p-values of the four regression models in each sample turned out to be highly significant (p<.001), meaning they would also remain significant after a potential correction for multiple testing.

Correlation coefficient of dependent variables - whole sample (N=322)

|  | PTSD symptoms | Depression symptoms | Internalizing problems | Externalizing problems |
| --- | --- | --- | --- | --- |
| PTSD symptoms | 1 | .68*** | .49*** | .38*** |
| Depression symptoms | .68*** | 1 | .62*** | .48*** |
| Internalizing problems | .49*** | .62*** | 1 | .51*** |
| Externalizing problems | .38*** | .48*** | .51*** | 1 |

***p≤.001

Correlation coefficient of dependent variables - RC (n=158)

|  | PTSD symptoms | Depression symptoms | Internalizing problems | Externalizing problems |
| --- | --- | --- | --- | --- |
| PTSD symptoms | 1 | .58*** | .54*** | .46*** |
| Depression symptoms | .58*** | 1 | .67*** | .58*** |
| Internalizing problems | .54*** | .67*** | 1 | .52*** |
| Externalizing problems | .46*** | .58*** | .52*** | 1 |

***p≤.001

Correlation coefficient of dependent variables - IDC (n=174)

|  | PTSD symptoms | Depression symptoms | Internalizing problems | Externalizing problems |
| --- | --- | --- | --- | --- |
| PTSD symptoms | 1 | .75*** | .44*** | .33*** |
| Depression symptoms | .75*** | 1 | .58*** | .40*** |
| Internalizing problems | .44*** | .58*** | 1 | .49*** |
| Externalizing problems | .33*** | .40*** | .49*** | 1 |

***p≤.001

**Editor Q3)**

**The reviewer also points out that the discussion considers rather tangential issues (e.g. possible intervention strategies) rather than providing more depth to the critical analysis.**

Thank you for also drawing our attention to the discussion. We have broadly expanded the limitations and rewritten the conclusions of our study by further elaborating on the methodological limitations of our data collection and the generalizability of the results.

*See manuscript page 19, line 450:*

*“Also, in terms of methodological limitations, a number of factors may have undermined the data collection process. Research in trauma-exposed children and adolescents is necessarily sensitive, so we have taken several precautions regarding participants wellbeing as well as data quality: First, all questionnaires were administered in the form of an interview. This also increased the reliability of the child's reports. However, research has shown that adding mother's reports could have specifically provided a more accurate picture on the experienced family violence (Hamby und Finkelhor 2020). Second, all interviewers received extensive training in psychological interviewing. Still, there may have remained some risk of interviewer bias by collecting data via interviews instead of self-report measures (Oh et al. 2018).”*

*See manuscript page 20, line 471:*

*“The present findings provide important evidence on forcibly displaced minors living in areas of ongoing or recent conflict, constituting the majority of displaced minors worldwide. The high rates of psychopathology observed confirm previous research in conflict-affected Iraq (Lafta und Al-Nuaimi 2019; Bolton 2013) and highlight the urgent need for mental health support for children and adolescents in this region.*

*Recommending specific interventions may be premature without further replication of our results. However, our findings support the potential benefits of a multi-level approach to addressing mental health needs in affected children and their families. Effective interventions for war-affected children, such as trauma-focused cognitive behavioural therapy (Chipalo 2021) and KidNET (Robjant und Fazel 2010), offer potential models for individual-level psychotherapy. Additionally, addressing family dynamics and community-level stressors may be important, but these recommendations should be approached with caution until further studies confirm their efficacy. Future research should also explore additional daily challenges faced by displaced minors and evaluate how post-migration conditions impact mental health. A holistic approach to interventions, incorporating these factors may ultimately prove beneficial.”*

**Editor Q4) I would also recommend you to significantly improve the quality of the figures as they are not readable.**

Thank you for pointing this out, you are right, we have corrected this mistake and the figures are now in good quality.

**Editor Q5)** **In any case, both the reviewer and I want you to have the opportunity to be published in Plos Mental Health after revising your work in the light of the major comments made by the reviewer and myself.**

Thank you for your positive feedback.

**Reviewer #1:**

**Thank you for the opportunity to review this manuscript. In the study presented, the authors highlight the different mental health burdens and corresponding predictive factors between refugee and internally displaced children. Although the range of variables interrogated was limited, this remains a valuable and rarely conducted comparison that should be of interest to many scholars. The authors are to be commended for the clear presentation of their study, although I suggest some amendments before publication, as listed below.**

**Introduction**
**The study frames trauma exposure as an individual-level factor which might be perplexing to some readers anticipating a Bronfenbrenner-informed approach. Individual-level factors usually encompass identities, traits, and personal characteristics, whereas trauma exposure (in this case, war exposure and displacement) might be better described as a community-level (exosystem) factor.**

Thank you for your comment. You are right that there has been inconsistency in how 'trauma exposure' is classified within the socioecological systems model (Bronfenbrenner) in previous literature. However, recent studies increasingly tend to classify individual exposure to war-related trauma as an individual-level factor (ontogenetic level). Community-level factors, in contrast, are more often associated with broader social influences like social support and neighbourhood quality. To align with this trend in the literature (Scharpf et al., 2021; Reed et al., 2012), we have chosen to conceptualize individual trauma exposure as an individual-level factor. Further, in the present study, trauma exposure was assessed using a checklist that includes various traumatic events, such as physical and sexual assault, bombings, car accidents, and life-threatening illnesses. We believe that the community-level aspect of these experiences is less relevant, as the nature and number of traumatic events experienced are ultimately highly individual.

Scharpf, F., Kaltenbach, E., Nickerson, A., & Hecker, T. (2021). A systematic review of socio-ecological factors contributing to risk and protection of the mental health of refugee children and adolescents. *Clinical Psychology Review*, *83*, 101930.

Reed, R. V., Fazel, M., Jones, L., Panter-Brick, C., & Stein, A. (2012). Mental health of displaced and refugee children resettled in low-income and middle-income countries: risk and protective factors. *The Lancet*, *379*(9812), 250-265.

**In the final paragraph of the introduction, the authors ask “Are mental health problems higher among IDC ID minors compared to RC, and do the same risk and protective factors contribute to the mental health of forcibly displaced IDC and RC?” I assume “IDC ID” is a typographical error?**

Thank you, you are right, we corrected this typographical error.

**Methods
Although detailed elsewhere, it may be worth reiterating the translation procedure for the study instruments, considering they had to be administered in two different languages.**

Thank you for this suggestion. You are right, the variety of languages spoken within our sample is indeed a factor worth describing further, which we did now.

*See manuscript page 7, line 160:*

*“While the key instruments had previously been developed and validated specifically for the study context in Arabic and Kurdish as described below (Ibrahim et al. 2021; Ibrahim et al. 2018), the remaining instruments were assessed using our own translations. Trained interviewers fluent in the local languages adapted the wording according to the specific individual language skills and preferences of each responding minor.”*

**In the statistical analysis section, the authors list the predictors chosen for multiple linear regression. The selected predictors, however, are only a subset of the variables reported in** **Table 1. Was there particular justification for excluding certain variables such as education, religion, ethnicity, place of living etc. from the regression analysis (especially considering the significant difference in some of these variables between IDC and RC)?**

Thank you for raising this topic. We explained the particular justification for excluding the variables reported in Table 1 in response to Editor’s Question 1 (Q1).

**While not mandatory, there may be merit in describing the “1.000 samples bootstrapping procedure” that was used, and why this was favoured over other procedures (e.g. variable transformation).**

Thank you for this recommendation. To our knowledge, this is the standard procedure recommended in the literature in the case of heteroscedasticity of the outcome variable in a multiple linear regression model, which we have now described in more detail, also adding a reference for more background information.

*See manuscript page 10, line 227:*

*“As the residuals for the outcome variables PTSD and depression deviated significantly from homoscedasticity, a 1.000 samples bootstrapping procedure, as a widely accepted method for addressing heteroscedasticity in multiple linear regression models (Astivia & Zumbo, 2019) was applied for these variables.”*

**Results
If my interpretation of the results is correct, a total of 12 regression models were fitted. Did these need to be corrected for multiple testing?**

Thank you for raising this important issue. We have carefully considered correcting for multiple testing, please see our reasoning in our response to the Editor's Q2.

**Discussion**
**- As part of the study limitations, the authors may wish to comment more on the data collection process (specifically, interviews with minors) and discuss what problems may have undermined this approach, especially given the sensitivity of some of the subject matter (e.g.** **interviewer bias; limited reliability of child-report etc). Consulting Oh et al., 2018 (**[**https://doi.org/10.1016/j.pedhc.2018.04.021**](https://doi.org/10.1016/j.pedhc.2018.04.021)**) may be helpful in this regard.**

Thank you for those ideas to deepen our discussion. We further elaborated on the truly sensitive characteristics of the data collection process by adding a paragraph in the limitations section of the paper.

*See manuscript page 19, line 451:*

*“Also, in terms of methodological limitations, a number of factors may have undermined the data collection process. Research in trauma-exposed children and adolescents is necessarily sensitive, so we have taken several precautions regarding participants wellbeing as well as data quality: First, all questionnaires were administered in the form of an interview. This also increased the reliability of the child's reports. However, research has shown that adding mother's reports could have specifically provided a more accurate picture on the experienced family violence (Hamby & Finkelhor, 2000). Second, all interviewers received extensive training in psychological interviewing. Still, there may have remained some risk of interviewer bias by collecting data via interviews instead of self-report measures (Oh et al., 2018).”*

**The authors should exercise caution in interchanging “multiple linear regression” with “multivariate regression” (e.g. see Hidalgo & Goodman, 2013, 10.2105/AJPH.2012.300897). On that note, as an additional sensitivity analysis and a way of reducing the total number of regression models (and risk of multiple testing), the authors might consider a multivariate regression model with all mental health outcomes as dependent variables in a single model.**

Thank you, you are right, we used the wrong expression here. We carefully considered multiple testing correction, please see our reasoning at Editor Q2).

**At times, the manuscript possibly reaches beyond its reasonable scope. For example, the discussion of intervention strategies may be premature, given that the study did not assess an intervention, and that the study results await replication in other samples.**

Thank you for your suggestion on how to improve the discussion. We have significantly shortened and revised our conclusions and have further emphasized the need for caution when generalizing the results of our study. However, based on our experience in humanitarian aid settings, we often observe that health agencies create intervention programs for mental health problems without a solid scientific evidence base. For this reason, we believe that the thorough examination presented in this study can still be valuable for humanitarian agencies as a basis for developing evidence-based interventions.

*“The present findings provide important evidence on forcibly displaced minors living in areas of ongoing or recent conflict, constituting the majority of displaced minors worldwide. The high rates of psychopathology observed confirm previous research in conflict-affected Iraq (Lafta und Al-Nuaimi 2019; Bolton 2013) and highlight the urgent need for mental health support for children and adolescents in this region.*

*Recommending specific interventions may be premature without further replication of our results. However, our findings support the potential benefits of a multi-level approach to addressing mental health needs in affected children and their families. Effective interventions for war-affected children, such as trauma-focused cognitive behavioural therapy (Chipalo 2021) and KidNET (Robjant und Fazel 2010), offer potential models for individual-level psychotherapy. Additionally, addressing family dynamics and community-level stressors may be important, but these recommendations should be approached with caution until further studies confirm their efficacy. Future research should also explore additional daily challenges faced by displaced minors and evaluate how post-migration conditions impact mental health. A holistic approach to interventions, incorporating these factors may ultimately prove beneficial.”*
