# Supplementary material for: Fleeing the war: A socio-ecological perspective on the mental health of internally displaced and refugee children and adolescents living in the Kurdistan region of Iraq
Source: PLOS Ment Health. 2025 Jan 30;2(1):e0000170. doi: 10.1371/journal.pmen.0000170 (PMC12798447; doi:10.1371/journal.pmen.0000170)
Supplement: Attachment — Submitted filename: Response to reviewers.docx [file pmen.0000170.s002.docx]

Dear Dr. Jacquet,

thank you very much for your positive feedback on the revision of our paper and especially for evaluating our revised manuscript yourself. As you suggested, we have now included further information on the exclusion of some predictors in the regression models, and hereby we resubmit the revised version of the manuscript.

Kind regards,

Jasmin Wittmann

*p. 10, line 230*

*We have selected the predictors based on the existing literature guiding our theoretical framework as well as our assessment of the interpretability of the variables. The following variables were not included in the regression models for the following reasons: Level of education: During data collection, we found that the interpretability of this variable (number of years of education) was limited. As there is no comprehensive, functioning school system in the camps, this variable is more related to factors such as age and gender, rather than reflecting the actual education level of the study sample. Religion and ethnicity:* *There is insufficient empirical evidence to support the hypothesis that mere religious or ethnic affiliation is independently associated with elevated mental health symptomatology. Consequently, we have refrained from positing such associations in our analysis. Place of residence during the war, place of residence before the war:* *We excluded these variables because they rely on retrospective information from several years ago. This does not reflect the children’s current living conditions, as they had been in the camp for an average of four years.*
